# Dysregulated autophagy increased melanocyte sensitivity to H_2_O_2_-induced oxidative stress in vitiligo

**DOI:** 10.1038/srep42394

**Published:** 2017-02-10

**Authors:** Yuanmin He, Shuli Li, Weigang Zhang, Wei Dai, Tingting Cui, Gang Wang, Tianwen Gao, Chunying Li

**Affiliations:** 1 Department of Dermatology, Xijing hospital, Fourth Military Medical University, Xi’an, Shaanxi, China

## Abstract

In vitiligo, melanocytes are particularly vulnerable to oxidative stress owing to the pro-oxidant state generated during melanin synthesis and to the genetic antioxidant defects. Autophagy is a controlled self-digestion process which can protect cells against oxidative damage. However, the exact role of autophagy in vitiligo melanocytes in response to oxidative stress and the mechanism involved are still not clear. To determine the implications of autophagy for melanocyte survival in response to oxidative stress, we first detected the autophagic flux in normal melanocytes exposure to H_2_O_2_, and found that autophagy was significantly enhanced in normal melanocytes, for protecting cells against H_2_O_2_-induced oxidative damage. Nevertheless, vitiligo melanocytes exhibited dysregulated autophagy and hypersensitivity to H_2_O_2_-induced oxidative injury. In addition, we confirmed that the impairment of Nrf2-p62 pathway is responsible for the defects of autophagy in vitiligo melanocytes. Noteworthily, upregulation of the Nrf2-p62 pathway or p62 reduced H_2_O_2_-induced oxidative damage of vitiligo melanocytes. Therefore, our data demonstrated that dysregulated autophagy owing to the impairment of Nrf2-p62 pathway increase the sensitivity of vitiligo melanocytes to oxidative stress, thus promote the development of vitiligo. Upregulation of p62-dependent autophagy may be applied to vitiligo treatment in the future.

Vitiligo is defined as a common depigmentary disease characterized by the destruction of melanocytes in the epidermis^1^. In both lesional and non-lesional skin of vitiligo patients, increased levels of reactive oxygen species (ROS) were observed, accompanied with reduced levels and activity of catalase^2^,^3^,^4^,^5^,^6^. It is well known that normal melanocytes are vulnerable to excessive ROS for their specialized function of melanin synthesis, which would result in a highly pro-oxidant state in the epidermis^7^,^8^. In fact, vitiligo melanocytes are particularly more sensitive to the accumulated ROS owing to the intrinsic antioxidant defects^8^^–^10. This imbalance between the pro-oxidants and the antioxidant status can disrupt the homeostasis of melanocytes, causing accumulation of oxidized and damaged proteins or organelles, and consequently lead to the destruction of melanocytes^7^,^11^.

Autophagy is a regulated cellular self-digestion process in which damaged intracellular proteins or organelles are sequestered and then transported to degrade in lysosomes for maintenance of cell homeostasis^12^. Interestingly, it has been discovered that constitutive autophagic activity is especially required to prevent oxidative damage and achieve the full proliferative capacity of normal melanocytes^13^. However, the exact role of autophagy in vitiligo melanocytes in response to oxidative stress has not been elucidated to date.

Nuclear factor E2-related factor 2 (Nrf2) is a critical transcription factor in protecting melanocytes from oxidative damage^14^. It is well established that Nrf2 exerts its protective effect by binding to the antioxidant response element (ARE) sequence and therefore promotes the expression of antioxidant genes^15^,^16^. Noteworthily, recent studies suggest that Nrf2-ARE pathway protects cells from various pro-oxidant stimuli via the induction of autophagy^17^,^18^. Thus, we speculated that Nrf2 serve as an essential upstream regulator of autophagy in stressed melanocytes. Our previous study has found vitiligo melanocytes showing functional defect in Nrf2-ARE pathway under the oxidative stress^10^. Hence, we further hypothesized that impaired autophagy caused by the dysregulation of Nrf2 pathway may increase the sensitivity of vitiligo melanocytes to oxidative stress and contribute to the pathogenesis of vitiligo.

To test our hypothesis, we first detected the autophagy flux in normal and vitiligo melanocytes exposure to H_2_O_2_. Then, we confirmed the protective effects of autophagy on melanocytes against H_2_O_2_-induced oxidative stress. Furthermore, we elucidated whether H_2_O_2_-induced Nrf2 activation contributes to autophagic activity. The underlying mechanisms were investigated as well.

## Results

### H_2_O_2_-induced oxidative stress increased autophagy flux in melanocytes

**Figure 1:**
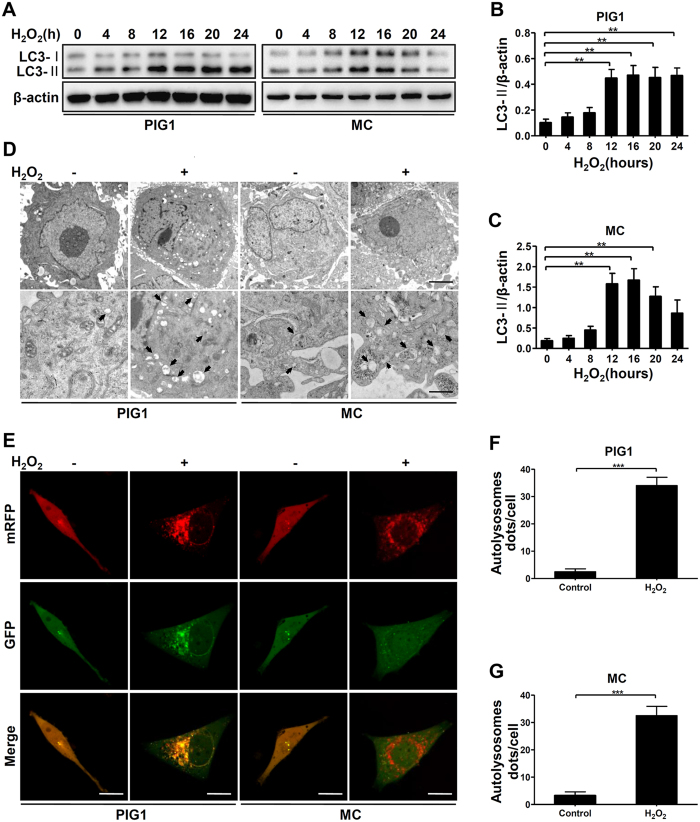
H_2_O_2_ treatment increases autophagy flux in melanocytes. (**A**) Immunoblots of LC3 in MC and PIG1 cells treated with H_2_O_2_ (0.5 mM) for different hours. (**B**,**C**) Band intensity normalized to β-actin is expressed as mean ± SD, **p < 0.01. (**D**) TEM images of autophagic vacuoles in cells treated with or without H_2_O_2_ for 12 hours. Arrows indicate the autolysosome. Scale bars represent 1 μm (upper row) and 500 nm (lower row). (**E**) MC and PIG1 cells were transfected with adenovirus expressing mRFP-GFP-LC3. After a 24-hour transfection, cells were treated with or without H_2_O_2_ for 12 hours. As shown in merged confocal images, the yellow puncta indicate the autophagosome while the red puncta indicate the autolysosome. Scale bars represent 5 μm. (**F**,**G**) The number of autolysosome dots were counted on 40 cells in a minimum of 3 experiments and expressed as mean ± SD, ***p < 0.001.

We firstly examined the autophagy levels of primary normal human melanocyte (MC) and the human melanocyte cell line (PIG1) treated with H_2_O_2_. We found the microtubule associated protein 1 light-chain 3 (LC3)-II, the most widely used biomarker of autophagosome formation, was significantly increased in MC and PIG1 cells following H_2_O_2_ treatment (Fig. 1A–C). And the conversion of LC3-I to LC3-II reached the highest level after 12 hours of H_2_O_2_ exposure in both groups (Fig. 1A–C). Subsequent ultrastructural transmission electron microscopy (TEM) analysis also showed that after exposure to H_2_O_2_ for 12 hours, MC and PIG1 cells displayed many cytoplasmic vesicles that had the typical single-membrane structure of autolysosome, whereas few autophagic vacuoles were observed in control cells (Fig. 1D). To further confirm the enhancement of autophagic flux induced by oxidative stress, mRFP-GFP-LC3 tandem construc was introduced. The results showed after exposure to H_2_O_2_ for 12 hours, both autophagosome (yellow puncta) and autolysosomes (red puncta) were increased in MC and PIG1 cells, whereas no significant changes were observed in control cells (Fig. 1E–G). These data demonstrated that H_2_O_2_-induced oxidative stress can induce autophagy in normal melanocytes.

### Autophagy protected melanocytes from oxidative stress-induced cell apoptosis, loss of mitochondrial membrane potential and ROS generation

**Figure 2:**
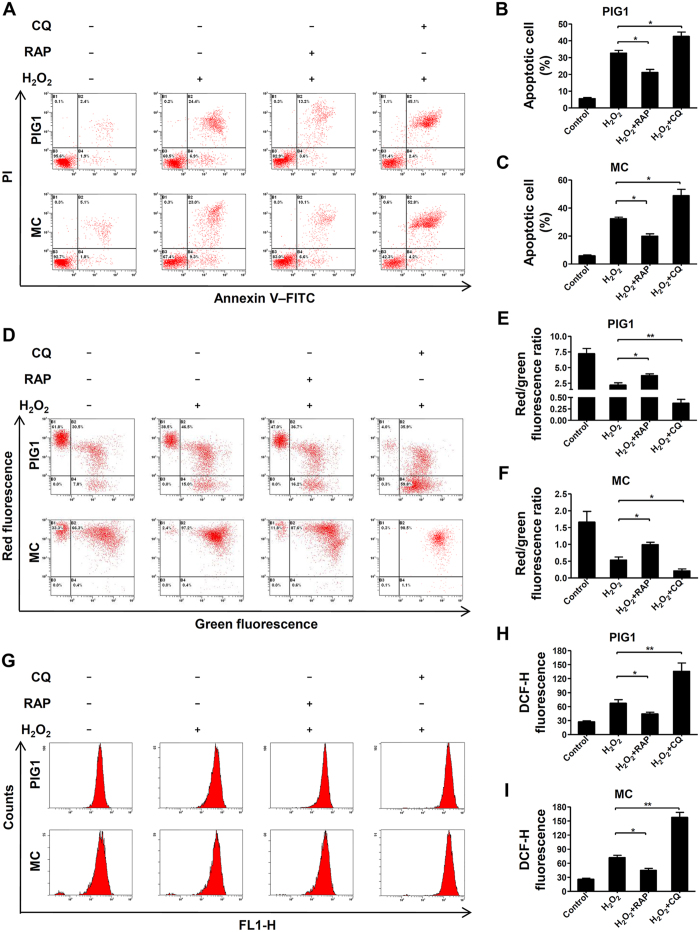
Increased autophagy protects melanocytes against oxidative stress while reduced autophagy renders cells more susceptible to oxidative stress. (**A**) MC and PIG1 cells were pretreated with rapamycin (RAP, 400 nM) or chloroquine (CQ, 50 μM) for 2 hours and treated with H_2_O_2_ (0.5 mM) for 24 hours, then the level of apoptosis was detected by flow cytometry assay. (**B**,**C**) The level of apoptosis are presented as the mean ± SD, *p < 0.05. (**D**) MC and PIG1 cells were pretreated with rapamycin or chloroquine for 2 hours and treated with H_2_O_2_ for 24 hours, then the MMP was measured by flow cytometry assay. (**E**,**F**) Quantification of MMP are presented as the mean ± SD, *p < 0.05, **p < 0.01. (**G**) MC and PIG1 cells were pretreated with rapamycin or chloroquine for 2 hours and treated with H_2_O_2_ for 24 hours, then the intracellular ROS level was quantized by flow cytometry assay. (**H,I**) Quantification of intracellular ROS level are presented as the mean ± SD, *p < 0.05, **p < 0.01.

We next identified the role of autophagy under oxidative stress in normal melanocytes. Cells were pretreated by rapamycin (RAP) to increase autophagic flux^19^, or chloroquine (CQ) to block autophagic activity^20^ in MC and PIG1 prior to treatment with H_2_O_2_. The results showed that addition of RAP significantly reduced cell apoptosis induced by H_2_O_2_, whereas CQ further promoted H_2_O_2_–induced apoptosis in both MC and PIG1 cells (Fig. 2A–C). Moreover, by using JC-1 dye and measuring the red/green ratio, a significant decrease in mitochondrial membrane potential (MMP) was detected in both MC and PIG1 cells following exposure to H_2_O_2_ for 24 hours, which were further promoted by CQ pretreatment, while the RAP rescued the cells from the loss of MMP (Fig. 2D). In addition, we investigated whether autophagy could eliminate excessive ROS induced by H_2_O_2_. As shown in Fig. 2E–G, the level of intracellular ROS induced by H_2_O_2_ was significantly attenuated when cells were pretreated with RAP, whereas CQ obviously increased H_2_O_2_-induced ROS generation. Taken together, these results confirmed that autophagy play a critical role in protecting normal melanocytes from oxidative stress-induced cell apoptosis, loss of MMP and intracellular ROS generation.

### Vitiligo melanocytes displayed impaired autophagy and increased sensitivity to H_2_O_2_-induced oxidative stress

**Figure 3:**
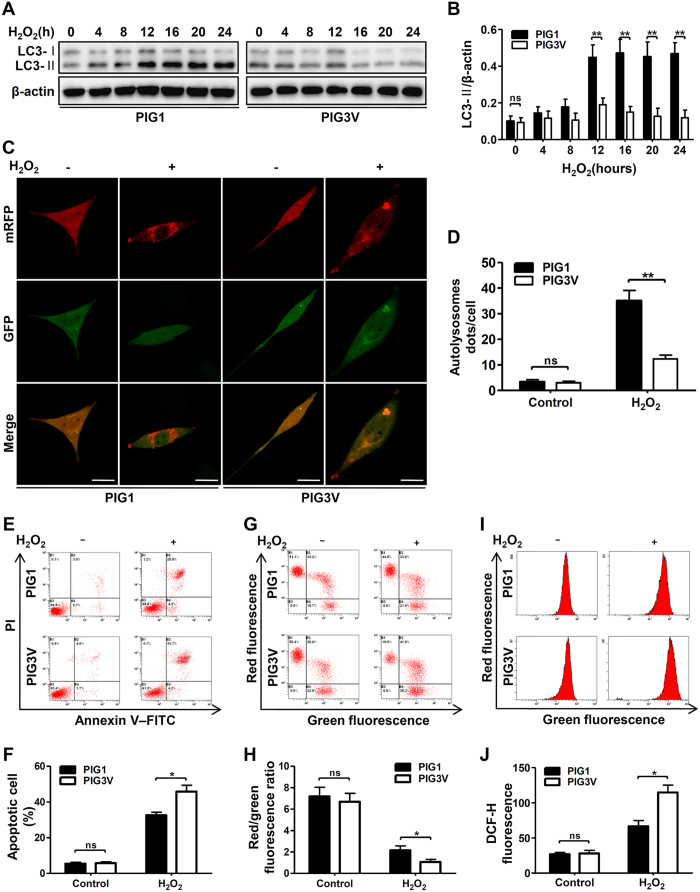
The difference in autophagy flux and cells sensitivity after H_2_O_2_ treatment between PIG1 and PIG3V cells. (**A**) Immunoblots of LC3 in PIG1 and PIG3V cells treated with H_2_O_2_ (0.5 mM) for different hours. (**B**) Band intensity normalized to β-actin is expressed as mean ± SD, **p < 0.01. (**C**) PIG1 and PIG3V cells were transfected with adenovirus expressing mRFP-GFP-LC3. After a 24-hour transfection, cells were treated with or without H_2_O_2_ for 12 hours. As shown in merged confocal images, the yellow puncta indicate the autophagosome while the red puncta indicate the autolysosome. Scale bars represent 5 μm. (**D**) The number of autolysosome dots were counted on 40 cells in a minimum of 3 experiments and expressed as mean ± SD, **p < 0.01. (**E**) PIG1 and PIG3V cells were treated with H_2_O_2_ for 24 hours, and the level of apoptosis was detected by flow cytometry assay. (**F**) The level of apoptosis are presented as the mean ± SD, *p < 0.05. (**G**) PIG1 and PIG3V cells were treated with H_2_O_2_ for 24 hours, and the MMP was measured by flow cytometry assay. (**H**) Quantification of MMP are presented as the mean ± SD, *p < 0.05. (**I**) PIG1 and PIG3V cells were treated with H_2_O_2_ for 24 hours, then the intracellular ROS level were measured by flow cytometry assay. (**J**) Quantification of intracellular ROS level are presented as the mean ± SD, *p < 0.05.

To evaluate whether autophagy was involved in the higher susceptibility to oxidative stress in vitiligo melanocytes, we first measured the level of autophagy in a vitiligo melanocyte cell line (PIG3V) exposed to H_2_O_2_. The results showed that after treatment with H_2_O_2_ for 12 hours, both the level of LC3-II and the ratio of LC3-II/-I were relatively lower in the PIG3V cells compared to that in PIG1 cells (Fig. 3A–C). According to mRFP-GFP-LC3 puncta formation assays, fewer autolysosomes dots in PIG3V cells were observed following H_2_O_2_ treatment for 12 hours than those of PIG1 cells (Fig. 3D). These observations suggested that vitiligo melanocytes have faint autophagic induction in response to H_2_O_2_-induced oxidative stress.

We further evaluated the effect of decreased autophagy in vitiligo melanocytes under oxidative stress by determine cell apoptosis, MMP and ROS generation. Compared to PIG1 cells, treatment of H_2_O_2_ caused more increased apoptosis (Fig. 3E,F), more reduced MMP (Fig. 3G,H), and more accumulated intracellular ROS (Fig. 3I,J) in PIG3V cells. Taken together, we concluded that vitiligo melanocytes with decreased autophagyic activity were more sensitive to H_2_O_2_-induced oxidative stress.

### Aberrant Nrf2-p62 signaling pathway is responsible for impaired autophagy in vitiligo melanocytes under oxidative stress

**Figure 4:**
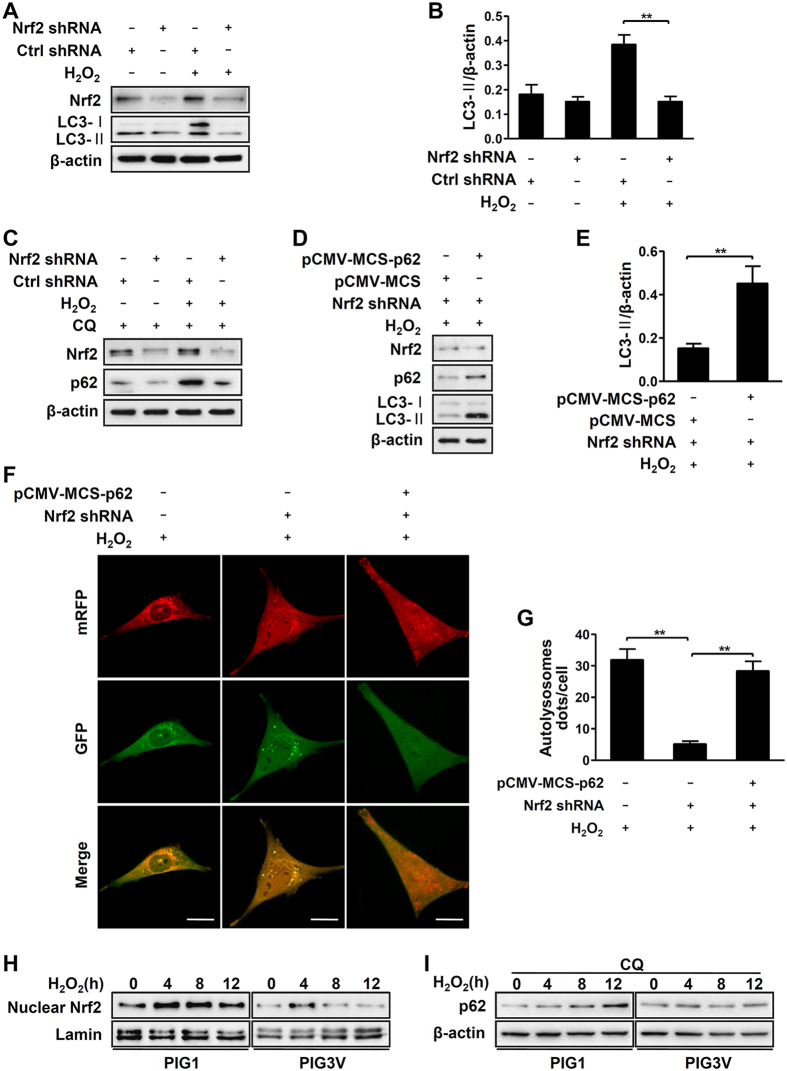
Nrf2-p62 pathway affected autophagy in melanocytes . (**A**) Immunoblots of Nrf2 and LC3 in PIG1 cells expressing Nrf2 shRNA, in the absence or presence of H_2_O_2_ (0.5 mM) for 12 hours. (**B**) The ratio of LC3-II/β-actin is presented as mean ± SD, **p < 0.01. (**C**) With chloroquine (CQ, 50 μM) pre-treatment, PIG1 cells expressing Nrf2 shRNA were treated with or without H_2_O_2_ for 12 hours. The expression level of Nrf2 and p62 were detected by western blotting. (**D**) PIG1 cells expressing Nrf2 shRNA were transfected with or without pCMV-MCS-p62 for 36 hours and then treated with H_2_O_2_ for 12 hours. The expression level of Nrf2, p62 and LC3 were detected by western blotting. (**E**) The ratio of LC3-II/β-actin is presented as mean ± SD, **p < 0.01. (**F**) PIG1 cells expressing Nrf2 shRNA were transfected with adenovirus expressing mRFP-GFP-LC3. After a 12-hour transfection, cells were treated with or without pCMV-MCS-p62 for 36 hours and then treated with H_2_O_2_ for 12 hours. As shown in merged confocal images, the yellow puncta indicate the autophagosome while the red puncta indicate the autolysosome. Scale bars represent 5 μm. (**G**) The number of autolysosome dots were counted on 40 cells in a minimum of 3 experiments and expressed as mean ± SD, **p < 0.01. (**H**) Immunoblots of nuclear Nrf2 in PIG1 and PIG3V cells treated with H_2_O_2_ for different hours. (**I**) Immunoblots of p62 in PIG1 and PIG3V cells co-treated with chloroquine and H_2_O_2_ for different hours.

Our previous study had ascertained that Nrf2 pathway has crucial role in protecting melanocytes from oxidative damage^14^. Meanwhile, recent studies have suggested that Nrf2 pathway protects cells from various pro-oxidant stimuli also via the induction of autophagy^18^. Therefore, we investigated whether Nrf2 pathway contributes to increase autophagy in normal melanocytes. Our results showed that the knockdown of Nrf2 by shRNA significantly attenuated the levels of LC3-II induced by H_2_O_2_ (Fig. 4A,B). Considering that Nrf2 contributes to the induction of p62/sequestosome1 which functions as an autophagic adaptor^21^, we examined whether the expression of p62 would be affected in normal melanocytes under H_2_O_2_-induced oxidative stress. Since p62 could also be degraded by autophagy, we detected the basic protein levels of p62 by addition of chloroquine which can block autophagic flux. As shown in Fig. 4C, Nrf2 knockdown blocked the expression of p62 in normal melanocytes. Furthermore, we restored the protein level of p62 in Nrf2 knockdown cells under oxidative stress, and observed that the declined LC3-II protein levels were rescued (Fig. 4D,E). Consistently, mRFP-GFP-LC3 puncta formation assays detected attenuated autolysosome puncta in Nrf2-knockdowned cells, whereas the number of the puncta significantly increased in these cells after over-expression of p62 (Fig. 4F,G). These data indicated that H_2_O_2_-induced autophagy can be regulated by the Nrf2-p62 pathway in normal melanocytes.

**Figure 5:**
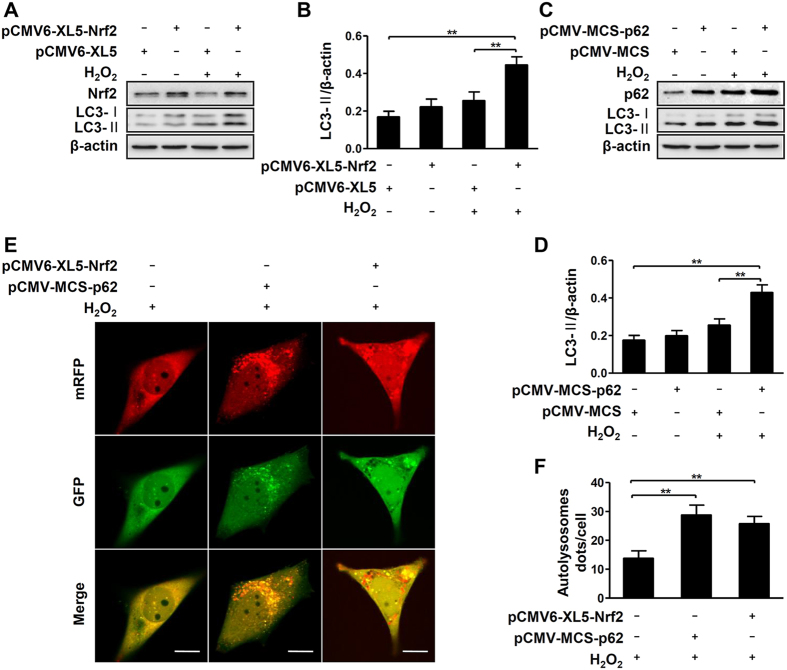
Upregulation of Nrf2 or p62 promotes H_2_O_2_-induced autophagy in vitiligo melanocytes. (**A**) Immunoblots of Nrf2 and LC3 in PIG3V cells which were transfected with pCMV6-XL5-Nrf2 for 36 hours and then were treated with H_2_O_2_ (0.5 mM) for 12 hours. (**B**) The ratio of LC3-II/β-actin is presented as mean ± SD, **p < 0.01. (**C**) Immunoblots of p62 and LC3 in PIG3V cells which were transfected with pCMV-MCS-p62 for 36 hours and then treated with H_2_O_2_ for 12 hours. (**D**) The ratio of LC3-II/β-actin is presented as mean ± SD, **p < 0.01. (**E**) PIG3V cells were transfected with adenovirus expressing mRFP-GFP-LC3. After a 12-hour transfection, cells were transfected with pCMV6-XL5-Nrf2 or pCMV-MCS-p62 for 36 hours and then treated with H_2_O_2_ for 12 hours. As shown in merged confocal images, the yellow puncta indicate the autophagosome while the red puncta indicate the autolysosome. Scale bars represent 5 μm. (**F**) The number of autolysosome dots were counted on 40 cells in a minimum of 3 experiments and expressed as mean ± SD, **p < 0.01.

We found both the nuclear Nrf2 translocation and basic protein levels of p62 failed to increase in response to H_2_O_2_-treated PIG3V cells compared to normal melanocytes (Fig. 4H,I). indicating an aberrant Nrf2-p62 signaling pathway in vitiligo melanocytes. Therefore, we further investigated whether the aberrant Nrf2-p62 pathway was the culprits for the damaged autophagy in vitiligo melanocytes under oxidative stress. Interestingly, upregulation of Nrf2 expression increased the protein levels of LC3-II in PIG3V cells after exposure to H_2_O_2_ (Fig. 5A,B). The increased LC3-II was correspondingly detected in p62-overexpressed PIG3V cells during oxidative stress (Fig. 5C,D). Moreover, increased autolysosome puncta were detected in both Nrf2- and p62-overexpressed PIG3V cells in response to H_2_O_2_ (Fig. 5E,F). Taken together, these results proved that aberrant Nrf2-p62 signaling pathway is responsible for impaired autophagy in vitiligo melanocytes under oxidative stress.

### Upregulation of p62 reduces cytotoxicity of vitiligo melanocytes under H_2_O_2_-induced oxidative stress

**Figure 6:**
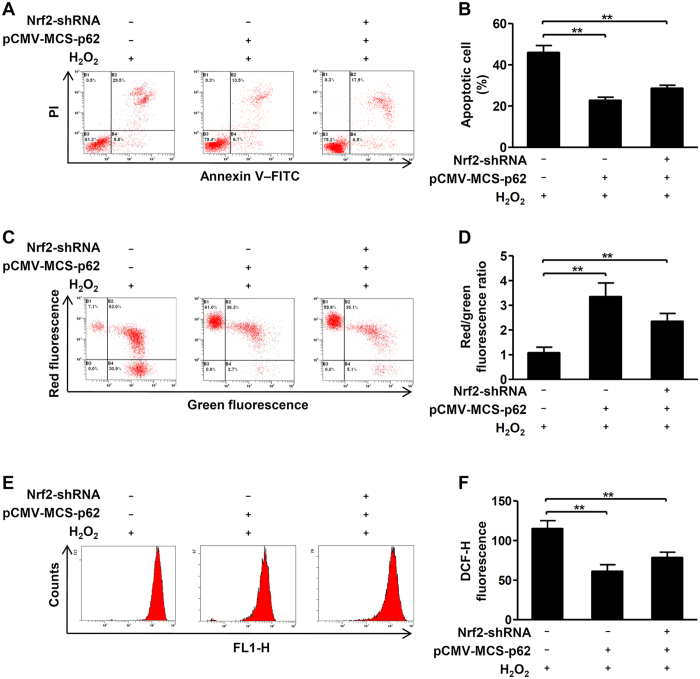
Upregulation of p62 reduces sensitivity of vitiligo melanocytes to H_2_O_2_-induced oxidative stress. (**A**) PIG3V cells were stably infected with or without the Nrf2 shRNA, then cells were transfected with or without pCMV-MCS-p62 for 24 hours and then treated with H_2_O_2_ (0.5 mM) for 24 hours, the level of apoptosis was detected by flow cytometry assay. (**B**) The level of apoptosis are presented as the mean ± SD, **p < 0.01. (**C**) PIG3V cells were stably infected with or without the Nrf2 shRNA, then cells were transfected with pCMV-MCS-p62 for 24 hours and then treated with H_2_O_2_ for 24 hours, the MMP was measured by flow cytometry assay. (**D**) Quantification of MMP are presented as the mean ± SD, **p < 0.01. (**E**) PIG3V cells were stably infected with or without the Nrf2 shRNA, then cells were transfected with pCMV-MCS-p62 for 24 hours and then treated with H_2_O_2_ for 24 hours, the intracellular ROS level was quantized by flow cytometry assay. (**F**) Quantification of intracellular ROS level are presented as the mean ± SD, **p < 0.01.

Finally, we try to investigate whether upregulation of p62 expression could reduce the cytotoxicity only by promoting autophagy in vitiligo melanocytes under oxidative stress. Of note, our results showed that upregulation of p62 significantly reduced cell apoptosis (Fig. 6A,B), restored the loss of MMP (Fig. 6C,D), and eliminated excessive intracellular ROS (Fig. 6E,F) in PIG3V cells and in Nrf2-knockdown cells treated with H_2_O_2_. All the above data demonstrated that even upregulation of p62 can independently protect vitiligo melanocytes against H_2_O_2_-induced oxidative stress.

## Discussion

**Figure 7:**
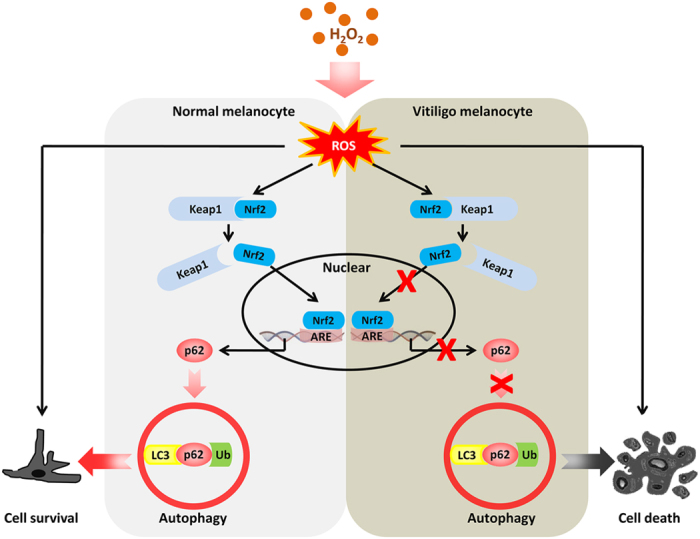
Nrf2-p62 pathway regulates autophagy in normal human melanocytes and vitiligo melanocytes under H_2_O_2_-induced oxidative stress. Under H_2_O_2_-induced oxidative stress in normal human melanocytes, Nrf2 dissociates from Kelch-like ECH-associated protein (Keap1) and then translocates to the nucleus where it binds to ARE and finally upregulates p62 expression. Upregulation of p62 can further promote cell autophagy and then protect melanocytes from H_2_O_2_-induced oxidative damage. On the contrary, in vitiligo melanocytes, impaired activation of the Nrf2-p62 pathway leads to decreased p62 expression, which fails to induce cell autophagy and then cause cell death exposed to H_2_O_2_-induced oxidative stress.

In this study, we firstly found that H_2_O_2_-induced oxidative stress enhances autophagic flux in primary human melanocytes against oxidative damage, and then established that Nrf2-p62 is an essential pathway in protecting melanocytes against H_2_O_2_-induced oxidative damage. Moreover, we confirmed that vitiligo melanocytes under H_2_O_2_-induced oxidative stress showed lower autophagic flux owing to the dysfunction of Nrf2-p62 pathway, which resulted in increased susceptibility to excessive ROS (Fig. 7). Giving the first evidence, our data pointed that the enhancement of autophagic activity is sufficient to reduce the oxidative damage in vitiligo melanocytes. Modification of autophagy may provide a promising therapy for vitiligo.

Autophagy is a key catabolic process that helps to maintain cell homeostasis under oxidative stress^19^,^22^,^23^. Because of the process of melanogenesis, excessive ROS is accumulated in melanocytes. Recently, Zhang *et al*. has reported their research on the autophagy process in melanocytes by detecting LC3, a classical protein marker for autophagy. Their study demonstrates that autophagy functions as a self-defense mechanism in mouse melanocytes against oxidative stress^13^. Since LC3 could be found in melanosomes, our study also used ultrastructural TEM to observe direct changes of autophagy flux in melanocytes, trying to exclude the interference of melanosomes when detecting autophagy using LC3 only. Noteworthily, the results of our TEM assay were always consistent with the ratio of LC3II/I detected by western blot in our study, indicating that using LC3 to detect autophagy in melanocytes is still feasible in spite of little expression of LC3 in melanosomes. Our results provide the first evidence that autophagy occurs to alleviate the oxidative damage as a part of cellular repair process in normal human melanocytes. Therefore, in addition to classical antioxidant system such as antioxidant genes or enzymes^7^, autophagy can protect melanocytes from oxidative stress, and consequently contribute to the cell homeostasis.

Our previous study has demonstrated that vitiligo melanocytes equipped with impaired antioxidant system, including the genetic defects of antioxidant enzymes^9^, dysfunctional Nrf2-HO1 antioxidant signaling pathway^10^, as well as aberrant expression of miRNAs^24^. In the current study, we confirmed that defects in autophagy could increase the apoptotic death and raise the intracellular ROS levels, as well as reducing the MMP in vitiligo melanocytes. These observations provide new evidences to explain why vitiligo melanocytes are more vulnerable to H_2_O_2_-induced oxidative damage compared to normal melanocytes.

Recently, several lines of evidence support an essential role for Nrf2 in the induction of autophagy, but the underlying mechanisms are still not fully understood^17^,^18^. It is well known that during autophagy, intracellular proteins and organelles can be banded to autophagosome by the adaptor protein p62^25^, which can be induced by the activation of Nrf2 under oxidative stress^21^. Moreover, Nrf2-mediated induction of p62 has been implicated in controlling Toll-like receptor-4 (TLR4)–driven selective autophagy structure formation and autophagic degradation^26^, as well as in cigarette smoke-induced autophagy in airway epithelial cells^17^. In this study, we also identified that Nrf2-mediated p62 upregulation is required for oxidative stress-induced autophagy in normal human melanocytes.

However, unlike normal melanocytes, both the activation of Nrf2 and the basic protein levels of p62 failed to increase in response to oxidative stress in vitiligo melanocytes. Notably, upregulation of Nrf2-p62 pathway increased the autophagic activity in vitiligo melanocytes, and therefore contributed to cell survival. These data suggested that the impairment of Nrf2-p62 pathway is responsible for the defects of autophagy in vitiligo melanocytes. Although several studies have reported that p62 could protect cells from oxidative damage via activation of Nrf2^27^,^28^, our current study demonstrated that even without activation of Nrf2, upregulation of autophagic flux by increasing p62 is sufficient to protect vitiligo melanocytes from H_2_O_2_-induced oxidative damage. These findings strengthen the importance of p62-dependent autophagy in melanocytes survival, providing a potential strategy for vitiligo treatment.

It has to be pointed out that only one vitiligo melanocyte cell line (PIG3V) was used in the current study. Considering that the genetic backgrounds vary among vitiligo patients, the cell line that we used can not completely represent the features of vitiligo melanocytes. Our findings need to be validated in other melanocyte lines extracted from vitiligo patients in future studies.

In conclusion, our study uncovers an essential role for autophagy in protecting normal human melanocytes from oxidative stress. Importantly, the dysregulation of Nrf2-p62 pathway causes the defects of autophagy under the condition of oxidative stress, which contributes to enhanced sensitivity of vitiligo melanocytes to oxidative stress. Our findings indicate a prospective treatment of upregulating p62-dependent autophagy for vitiligo.

## Materials and Methods

### Cell culture and reagents

Primary normal human melanocytes were isolated from human foreskin specimens obtained during circumcision surgery. The primary melanocytes were grown in Medium 254 (Cascade Biologics, Portland, OR, USA) containing Human Melanocyte Growth Supplement (Cascade Biologics). The primary melanocytes were used between 2^nd^ and 4^th^ passage in all experiments. The immortalized normal human epidermal melanocyte cell line PIG1 and a vitiligo melanocyte cell line PIG3V (both gifts from Dr. Caroline Le Poole, Loyola University Chicago, Maywood, IL, USA) was cultured in Medium 254 (Cascade Biologics) containing Human Melanocyte Growth Supplement (Cascade Biologics) and 5% fetal bovine serum (Invitrogen, San Diego, CA, USA). Cells were maintained at 37 °C in a humidified atmosphere containing 5% CO_2_. H_2_O_2_ (Sigma-Aldrich, St Louis, MO, USA) was used at concentration of 500 μM. Rapamycin (Abcam, Cambridge, UK) was used at concentration of 400 nM. Chloroquine (Sigma-Aldrich) was used at concentration of 50 μM. All subjects consented by written and informed agreement for inclusion in this study. All experiment protocols were approved by the Ethics Committee of the Fourth Military Medical University, in accordance with the Declaration of Helsinki principles.

### Lentiviral infection

Cells were infected with Nrf2 shRNA (5′-GGGAGGAGCTATTATCCATTC-3′, Genepharma, Shanghai, China) or Ctrl shRNA (Genepharma, Shanghai, China) at a 1:4 dilution in the presence of 5 μg/mL polybrene (Genepharma, Shanghai, China). After 24 h, cells harboring the Nrf2 shRNA or Ctrl shRNA cassettes were selected in the presence of puromycin (0.3 μg/ml; abcam) for 3 days. Expression of the constructs was confirmed by western blotting.

### Plasmids and transfection

Cells were seeded at 2 × 10^5^ cells per well for 24 hours before transfection. Cells were transfected with pCMV6-XL5-Nrf2 (OriGene, MD, USA) or pCMV-MCS-p62 (Genechem, Shanghai, China) at 4 mg per well for 6-well plates by Lipofectamine 3000 (Invitrogen). After 48-hour incubation, cells were assessed protein changes. The pCMV6-XL5 or pCMV-MCS transfection and control cells (no transfection) were included in each experiment.

### Western blot

Cells were washed with phosphate buffered saline (PBS) and lysed in RIPA buffer (Runde Biologicals, Xi’an, China) containing phenylmethanesulfonyl fluoride (protease inhibitor mix; Sigma-Aldrich). Equal amounts of the protein samples were separated on a 12% sodium dodecyl sulfate-polyacrylamide gel and blotted onto a nitrocellulose membrane (Millipore, Bedford, MA, USA). After blocking by 5% non-fat dry milk, the membranes were washed and incubated with primary antibodies overnight at 4 °C. After washing, the membranes were incubated with horseradish peroxidase–conjugated secondary antibodies for 1 hour at room temperature. Bound antibodies were detected using a chemiluminescence detection kit (KPL, Gaithersburg, MD). Primary antibodies against LC3 I/II and p62 were purchased from Cell Signaling Technology, Inc., Danvers, MA; Antibodies against Nrf2 were purchased from Abcam, MA; Anti-Lamin and anti-β-actin were purchased from Life Science Products &Services, shanghai, China. Second antibodies including anti-mouse IgG or anti-rabbit IgG were purchased from Santa Cruz. Band intensities were determined by software (ImageJ; National Institutes of Health, Bethesda, MD).

### Detection of apoptosis

After experimental treatment, cells were detected by the Annexin V/PI Apoptosis Detection Kit (MaiBio, Shanghai, China) following the manufacturer’s instructions and identified in flow cytometer (Beckman Coulter, Miami, USA) within an hour. The analysis was done with Expo32 software (Beckman Coulter).

### Assay for ROS levels

After experimental treatments, cells were washed with PBS and incubated in PBS containing 10 mM CM-H_2_DCFDA (Invitrogen) for 30 min at 37 °C in the dark. Then cells were washed twice in PBS, and fluorescence was measured by flow cytometer (Beckman Coulter) within an hour. Mean fluorescence intensity was quantized with Expo32 software (Beckman Coulter).

### Measurement of mitochondrial membrane potential

After experimental treatments, cells were washed with PBS and loaded with JC-1 dye (Invitrogen) 5 mg/mL for 20 min at 37 °C. Then cells were washed twice in PBS, and fluorescence was measured by flow cytometer (Beckman Coulter) within an hour. Mean fluorescence intensity was quantized with Expo32 software (Beckman Coulter) and red/green ratio was calculated as an indicator of mitochondrial membrane potential.

### Transmission electron microscopy

After experimental treatments, cells were collected and washed in cold PBS, fixed in fixative buffer for 24 hours at 4 °C. Then the cell pellet was rinsed with Millonig’s buffer and minced post-fixed in 1.0% OsO_4_, The pellet was stained with 2.0% aqueous uranyl acetate followed by ethanol dehydration, and embedded in Spurr’s plasticresin. Ultrathin section analysis was visualized using a Tecnai™ G2 Spirit Bio Twin (FEI, Hillsboro, OR).

### Tandem mRFP-GFP-LC3 fluorescence microscopy

Cells were transfected with adenovirus expressing mRFP-GFP-LC3 (Hanbio, Shanghai, China) at 10 MOI for 24 h, and then treated with or without other agentia. The cells were analyzed by confocal laser scanning microscopy (LSM510; Carl Zeiss AB, Jena, Germany).

### Statistical analysis

Each experiment was performed at least for three times, and statistical analyses of the data were performed using unpaired, two-tailed Student’s t-tests or using one-way analysis of variance (ANOVA), followed by Newman-Keuls test built into GraphPad Prism (GraphPad Software 5.0; San Diego, CA). All the data were expressed as mean ± SD. Differences with P < 0.05 were considered statistically significant.

## Additional Information

**How to cite this article**: He, Y. *et al*. Dysregulated autophagy increased melanocyte sensitivity to H_2_O_2_-induced oxidative stress in vitiligo. *Sci. Rep.* **7**, 42394; doi: 10.1038/srep42394 (2017).

**Publisher's note:** Springer Nature remains neutral with regard to jurisdictional claims in published maps and institutional affiliations.
